# Insights Into Skin-Lightening Practices of Hijra and Transgender Communities in India

**DOI:** 10.2196/66822

**Published:** 2025-05-29

**Authors:** Sriram Palepu, Vasudeva Murthy Sindgi, Aylur Kailasom Srikrishnan, Carrie Kovarik

**Affiliations:** 1 Perelman School of Medicine, Philadelphia, PA, United States; 2 Jayamukhi College of Pharmacy, Warangal, India; 3 YR Gaitonde Centre for AIDS Research and Education, Chennai, India; 4 Department of Dermatology, University of Pennsylvania, 3600 Spruce St., Philadelphia, PA, 19104-5162, United States, 1 215-662-2737

**Keywords:** skin lightening, India, medication misuse, insight, hijra, transgender women, treatment, patient education, skin care, community, fairness cream, marketing, ads, advertisement, cost, lightening cream, cross-sectional study, survey study

## Abstract

A large proportion of transgender women in Hyderabad, India (150/223, 67.3%) expressed interest in a wide range of topical, oral, and intravenous medications for skin lightening; however, despite government regulations and the potential health risks, persistent demand for skin lightening underscores the need for better patient education and safer skin care practices for this marginalized community.

## Introduction

The Government of India’s recent amendments to the Drugs and Magic Remedies Act proposed increased penalties for marketing and advertising skin fairness creams [1]. Yet, conventional Indian beauty standards still drive demand for skin-lightening products (SLPs) among women in India and transfeminine communities. Literature on skin-lightening practices among transgender people is limited. Transgender women undergoing estrogen therapy have an increased risk of melasma, which may be treated with skin-lightening agents like hydroquinone [2]. An ethnographic study of Indonesia’s transfeminine *waria* community found that members sought SLPs to feel more feminine and attract male attention [3]. Similar motivations were documented among Thailand’s transgender entertainers [4].

The Health Needs and Aesthetic Preferences Assessment of the Hyderabad Trans Community is a large study evaluating the social and health history of transgender and *hijra* women in India (N=300). As part of that study, we evaluated the prevalence of interest in skin-lightening treatments, the products used, and the financial costs involved.

## Methods

### Study Design

This cross-sectional survey study was conducted at Mitr Clinic (Hyderabad, India), using consecutive sampling. The survey was developed by the research team and administered in Hindi, Telugu, or English. The inclusion criteria were as follows: *hijra* and/or transgender women aged ≥18 years, seeking female gender affirmation, and South Asian ancestry. Transgender men, individuals seeking male gender affirmation, and individuals aged <18 years were excluded. Dollar amounts were estimated based on the March 19, 2023, exchange rate.

### Ethical Considerations

Because some community members have limited literacy, verbal consent was obtained before data collection. No protected health information was collected. Institutional review board (IRB) approvals were obtained from the University of Pennsylvania and YR Gaitonde Centre for AIDS Research and Education—the clinic’s overseeing nonprofit. Remuneration (₹500 [US $5.84]) was provided to patients for their time and participation.

## Results

An IRB addendum approved in December 2023 enabled 74.3% (223/300) of participants to respond to skin lightening–related questions. More than two-thirds (150/223, 67.3%) of respondents expressed interest in skin lightening, of whom 43.3% (65/150) used SLPs. The overall prevalence of SLPs among respondents was 29.1% (65/223). Further, 1.3% (2/150) of respondents used SLPs previously but lost interest, and 3.1% (7/223) could not recollect or identify the products they used. Money spent on skin lightening varied from ₹25 (US $0.30) to ₹70,000 (US $843; median ₹570 [US $7], IQR ₹2225 [US $27]). Patients sometimes used multiple products (Table 1).

**Table 1.** Ingredients of self-reported products used for skin lightening.

Type of product used^a^	Active ingredients in products^b^
Topical treatments (n=23)	Hydroquinone + tretinoin + mometasone furoate (n=15) Clobetasol + neomycin + miconazole nitrate (n=1) Glycolic acid + arbutin + kojic acid (n=1) Terbinafine + ornidazole + oflaxicin + clobetasol (n=2) Betamethasone cream (n=3) Sunscreen (n=1)
Oral medications (n=2)	Levonorgestrel/ethinyloestradiol (n=1) Biotin + multivitamin (n=1)
Intravenous medications (n=16)	Glutathione (n=16)
Alternative (herbal/Ayurvedic/Unani; n=5)	Combination of ingredients, including lycopene, botanical extracts (eg, mallow, cowslip, licorice, and aloe vera), and soy isoflavones
Marketed beauty creams (n=15)	Combination of ingredients, including herbal ingredients, kojic acid, niacinamide, vitamin C, vitamin E, and sun protection factors (octocrylene, avobenzone, etc)

a The n values in this column refer to the number of products reported.

b The n values in this column refer to the number of products that contained the active ingredients listed in this column.

## Discussion

Our study highlights the considerable interest in SLPs but marginally low prevalence of SLP use (likely due to financial barriers) among *hijra* and transgender women. Survey studies on cisgender populations in India indicate that SLPs have widespread prevalence (range 34%-60%) [5,6]. Deeply rooted cultural norms associate lighter skin with economic prosperity and beauty, leading to widespread use even among South Asian immigrant communities [7]. Although 67.3% of our respondents expressed interest in skin lightening, only 29.1% used SLPs—a lower rate than in cisgender communities. Within the colorism context, transfeminine individuals may view skin lightening as an accessible method for facilitating gender affirmation and social acceptance, as observed in other Asian countries.

Transgender women often face stigma and discrimination in health care settings, preventing them from seeking care [8]. Additionally, many transgender women in India engage in sex work, which acts a strong economic driver for investing in physical appearance (eg, undergoing skin treatments to achieve a desired aesthetic) [8]. Although many respondents were interested in skin lightening, less than half used SLPs, which included herbal mixtures from local shops and intravenous glutathione injections.

A study on SLPs used in India reported topical medication misuse prior to seeing a dermatologist [9]. The combination of hydroquinone, mometasone, and tretinoin cream is a common, over-the-counter melasma treatment in India [9]. Glutathione injections, though popular and expensive, have questionable efficacy [10]. Some alternative, traditional remedy–based medications are often cheaper. Popular marketed beauty creams use ingredients like kojic acid, niacinamide, and arbutin, which have been studied for their effects on skin pigmentation and complexion [11]. Chronic steroid use, while lightening some patients’ skin, may result in skin atrophy and other side effects [12]. Antifungal creams may treat pigmentary changes resulting from infections like pityriasis versicolor but have no additional lightening effects. Only 1 respondent reported using sunscreen daily, beyond sun protection factors in beauty creams.

Almost half of our respondents use medications with skin-lightening properties—mostly purchased over the counter. After the COVID-19 pandemic, the average *hijra* community member’s earnings decreased from US $7 to US $13 per day to less than US $2 per day [13]. Many members are of low socioeconomic status and have been reported to seek hormonal and surgical care from unqualified medical practitioners because allopathic treatments are costly [14]. Despite the Indian government’s regulatory efforts, interest in SLPs persists among transgender women [1]. However, only a fraction can afford to regularly use skin-lightening treatments. Given the potential health and financial risks, patient education about safe skin care is crucial for transgender women to make informed health decisions.

This study had several limitations, which we hope to address in follow-up studies. Participants’ informal occupations (eg, begging and sex work) precluded an accurate income assessment. Furthermore, the ad hoc survey lacked prior psychometric validation; this may have affected the accuracy of estimates regarding SLP use. Lastly, data on individual product costs and usage durations were not collected, limiting insights into the costs of long-term use.

## Abbreviations

IRB: institutional review board
SLP: skin-lightening product
